# Rapid test detection of anti-infliximab antibodies: performance comparison with three different immunoassays

**DOI:** 10.1177/1756284820965790

**Published:** 2020-11-18

**Authors:** Cátia Rocha, Paula Lago, Samuel Fernandes, Luís Correia, Francisco Portela, Ana Isabel Vieira, Marta Patita, Bruno Arroja, Paula Ministro, Catarina Alves, Cláudia Camila Dias, Fernando Magro

**Affiliations:** Department of Biomedicine, Unit of Pharmacology and Therapeutics, Faculty of Medicine, University of Porto, Porto, Portugal; Faculty of Medicine, University of Lisbon, Lisbon, Portugal; Institute of Environmental Health, Faculty of Medicine, University of Lisbon, Lisbon, Portugal; Department of Gastroenterology, Centro Hospitalar do Porto, Porto, Portugal; Department of Gastroenterology and Hepatology, Centro Hospitalar Lisboa Norte, Hospital de Santa Maria, Lisbon, Portugal; Department of Gastroenterology and Hepatology, Centro Hospitalar Lisboa Norte, Hospital de Santa Maria, Lisbon, Portugal; Department of Gastroenterology, Centro Hospitalar e Universitário de Coimbra, Coimbra, Portugal; Department of Gastroenterology, Hospital Garcia de Orta, Almada, Portugal; Department of Gastroenterology, Hospital Garcia de Orta, Almada, Portugal; Department of Gastroenterology, Hospital de Braga, Braga, Portugal; Department of Gastroenterology, Centro Hospitalar Tondela-Viseu, Viseu, Portugal; Faculty of Medicine, University of Porto, Porto, Portugal; Center for Health Technology and Services Research (CINTESIS), Faculty of Medicine, University of Porto, Porto, Portugal; Health Information and Decision Sciences Department, Faculty of Medicine, University of Porto, Porto, Portugal; Department of Biomedicine, Unit of Pharmacology and Therapeutics, Faculty of Medicine, University of Porto, Alameda Prof. Hernâni Monteiro, Porto, 4200-319, Portugal; Portuguese IBD Study Group (GEDII), Porto, Portugal; Department of Gastroenterology, São João Hospital Centre, Porto, Portugal

**Keywords:** antibodies, drug monitoring, enzyme-linked immunosorbent assay, immunoassay, inflammatory bowel diseases, infliximab, point-of-care systems

## Abstract

**Background and Aims:**

Therapeutic drug monitoring (TDM) of infliximab (IFX) and anti-infliximab antibodies (ATIs) is essential for treatment optimisation in inflammatory bowel disease (IBD) patients. The aim of this study was to estimate and compare the agreement and accuracy between a new rapid test and three established enzyme-linked immunosorbent assays (ELISAs) to quantify ATIs levels, and to evaluate the impact of exogenous IFX on the performance of these assays.

**Methods:**

We analysed 200 serum samples from 57 IBD outpatients in IFX induction or maintenance therapy at six IBD centres in Portugal. ATI levels were quantified using the rapid test Quantum Blue® (QB) Anti-Infliximab (Bühlmann) and three established ELISAs: In-House, Theradiag (Lisa Tracker Anti-Infliximab), and Immundiagnostik (IDKmonitor Infliximab). ATIs were quantified in patients’ serum samples and spiked samples with exogenous IFX, based on analytical and clinical cutoffs. Qualitative agreement and accuracy were estimated by Cohen’s kappa (*k*) with 95% confidence intervals.

**Results:**

ATIs quantification with clinical cutoffs showed a slight agreement between QB rapid test and In-House [*k* = 0.163 (0.051–0.276)] and Immundiagnostik [*k* = 0.085 (0.000–0.177)]. Regarding IFX/ATIs status, the QB rapid test showed a substantial agreement with Theradiag [*k* = 0.808 (0.729–0.888)] and a fair agreement with In-House [*k* = 0.343 (0.254–0.431)] and Immundiagnostik [*k* = 0.217 (0.138–0.297)]. The QB rapid test could not detect ATI-positive levels in samples with exogenous IFX at 5–300 µg/ml. Interference on ATIs detection was observed at exogenous IFX ⩾30 µg/ml for In-house and Immundiagnostik assays.

**Conclusion:**

QB rapid test is only suitable to detect ATI-positive levels in the absence of IFX.

## Introduction

Infliximab (IFX) is a therapeutic monoclonal antibody against tumour necrosis factor alpha (TNF-α). IFX is effective in lowering disease activity and inducing clinical remission in patients with inflammatory bowel disease (IBD).^1^ However, up to 30% of patients fail to respond to induction treatment (primary loss of response), and 50% of patients may lose response during maintenance treatment (secondary loss of response), many during the first year.^2,3^ This loss of response to IFX therapy may occur due to several reasons, including the development of anti-drug antibodies (ADAs).^4^ ADAs may neutralise drug-target binding and increase drug clearance, resulting in suboptimal concentrations of active drug and shorter duration of response.^5–7^ Previous studies have shown that up to 44% of IBD patients treated with IFX develop anti-IFX antibodies (ATIs), depending on dosing schedules, concomitant use of steroids or immunomodulators, and measurement methods.^8–12^ When loss of response occurs, physicians usually change the therapeutic strategy by increasing the dosage or frequency of the current drug therapy, switch to another TNF-α antagonist, or switch to a different class of drug with another mode of action.^13^ However, this empirical approach increases the risk of irreversible tissue damage and healthcare costs, and could delay effective IBD treatment.^1,14^ Therefore, the assessment of drug and ADA levels, also known as therapeutic drug monitoring (TDM), is essential to define future therapeutic strategies. The specific assessment of IFX and ATI levels allows physicians to understand the reasons for unresponsiveness, identify patients that will most benefit from the dose adjustment of current IFX therapy or from switching to another drug,^7,14,15^ and reduce delays in effective treatment.^1,14^ TDM is thus essential to define therapeutic strategies in IBD patients, improving clinical outcomes and minimising IBD-related complications.

TDM has led to the development of methods for quantification of IFX and ATI levels with different applications and limitations. Of particular concern is that some methods quantify both IFX and ATIs, whereas others are specific for only one of these quantifications, which may have a significant impact on TDM’s results and interpretation.^3,16,17^ Several commercial kits measure IFX levels in the patient’s serum, most of them relying on enzyme-linked immunosorbent assays (ELISAs).^16,18,19^ However, these assays have a turnaround time of approximately 8 h, which might impair immediate adjustment of IFX therapy. In contrast, recent developments in rapid point-of-care tests allow the semi-quantitative assessment of ATI levels from the patient’s serum within minutes.^20,21^ In fact, the recent development of a rapid test to ATIs quantification (Quantum Blue® Anti-Infliximab, Bühlmann) promise a fast detection of ATIs (15–20 min turnaround time) on a single sample.^20^ These assays facilitate TDM and immediate adjustment of the IFX dosage. Nevertheless, the use of point-of-care tests for the quantification of ATIs in clinical practice is still limited by a lack of data and there is a need to evaluate their inter-assay heterogeneity and accuracy.^22^ Also, the impact of detectable IFX levels in patients’ serum on the TDM is understudied, particularly for the quantification of ATI levels by already established ELISAs.^3,16,17,23^ This indicates a need to evaluate and compare the performance of the various assays currently used in clinical practice to quantify ATI levels, to improve clinical decision-making based on TDM.

With this study we aimed to estimate and compare the accuracy and agreement between a new rapid test and three different established ELISAs for quantifying ATI levels in the serum of IBD patients. We also aimed to evaluate the impact of exogenous IFX on the performance of the four assays. We selected the recently commercially available rapid test Quantum Blue® Anti-Infliximab (Bühlmann) and the established In-House, Lisa Tracker Anti-Infliximab (Theradiag), and IDKmonitor Infliximab (Immundiagnostik) assays for quantification of ATI levels using analytical or clinical cutoff levels.

## Methods

### Patients and sample collection

This was a multicentre, non-interventional, retrospective study. From July 2016 to August 2019, 200 clinical samples were collected at six IBD centres in Portugal from 57 IBD patients attending routine outpatient consultations. The study population comprised patients who were adults (⩾18 years), male or female, diagnosed with moderate-to-severe active Crohn’s disease or ulcerative colitis, primary responders to IFX induction doses were assessed clinically and endoscopically, and received at least three IFX maintenance doses.

The clinical samples were obtained from patients undergoing the induction or maintenance treatment phase, and immediately before the infusion of a new IFX dose. Collected baseline sociodemographic and clinical data included birth date, date of diagnosis, sex, smoking status, diagnosis of Crohn’s disease or ulcerative colitis, and concomitant IBD-related medication. Blood samples were collected, centrifuged, and serum samples were kept at −80°C until being processed.

Potentially eligible samples were identified based on the previous quantification of ATI levels in our laboratory using our reference method (In-House assay). Samples were consecutively chosen to cover clinically relevant cutoff points for ATI negativity (<1.7 μg/ml) and positivity (⩾1.7 μg/ml) defined in the literature.^17,24^ ATI samples were included according to the following cutoff levels: negative, <1.7 μg/ml; low, 1.7–2.9 µg/ml; intermediate, 3.0–9.9 μg/ml; and high, ⩾10 μg/ml. Trough IFX concentrations were previously measured for all samples as part of the clinical routine using Quantum Blue® Infliximab (Bühlmann, Schönenbuch, Switzerland). More detailed information about the assays and protocols can be found in Supporting Information The study was conducted in accordance with the Declaration of Helsinki and was approved by the Ethics Committees of each centre. All patients signed a written informed consent before their participation.

### Quantification of ATI levels

All samples were analysed with the rapid point-of-care test Quantum Blue® Anti-Infliximab assay (Bühlmann, Schönenbuch, Switzerland), hereafter referred to as QB rapid test, according to the manufacturers’ instructions (Supporting Information). The ATI levels were calculated as IgG equivalents to the monoclonal reference antibody used for standardisation (μg_eq_/ml) and hereafter expressed as μg/ml. The following three ELISAs were used as comparators: In-House assay, Lisa Tracker anti-Infliximab (Theradiag, Croissy Beaubourg, France), and IDKmonitor Infliximab total ADA ELISA (Immundiagnostik, Bensheim, Germany). The quantifications using Theradiag and Immundiagnostik were performed following manufacturers’ instructions,^25–27^ whereas the In-House assays were carried out as previously described by Ben-Horin *et al.*^17,28^ (Supporting Information). The lower and upper detection limits for ATI levels described by the manufacturers were as follows: QB rapid test, 0.6–12 μg/ml; In-House assay, lower limit of 1.2 μg/ml; Theradiag, 0.01–0.2 μg/ml; and Immundiagnostik, higher average optical densities >10 antibody units (AU)/ml were classified as positive. All kits and samples were used and processed by the same technician.

Because the four assays tested have different technical characteristics, detection limits and expression of results, the test positivity cutoffs for a qualitative evaluation of the ATI levels are difficult to establish. Therefore, we used analytical and clinical cutoffs to test ATI-positive (ATI+) levels. The analytical cutoffs were based on the lower detection limits described by the manufacturers for each assay, while the clinical cutoffs used clinically relevant ATI+ levels defined in the literature.^17,24^ Using the analytical cutoffs, ATI+ levels were defined as ATI levels ⩾0.6 μg/ml for QB rapid test, ⩾1.2 μg/ml for In-House, ⩾0.01 μg/ml for Theradiag, and ⩾10 AU/ml for Immundiagnostik. Using the clinical cutoffs, ATI+ levels were defined as ATI levels ⩾1.7 µg/ml for QB rapid test, In-House and Theradiag, and ⩾10 AU/ml for Immundiagnostik.

### Exogenous IFX in ATI-positive serum samples

To assess the impact of IFX in the quantification of ATI levels, exogenous IFX (Schering Plough, New Jersey, USA) was added to ATI+ serum samples with undetectable IFX concentrations (IFX–) <0.4 μg/ml. ATI+ serum samples with low, intermediate, and high ATI levels were selected – six different samples were selected for each ATIs group. Serum samples with ATI+ levels and IFX- concentrations were preincubated with several exogenous IFX concentrations (5, 10, 15, 30, 100 and 300 μg/ml) for 30 min at room temperature, as previously described by our group.^23^ The therapeutic range of IFX concentrations was considered to be between 0 and 100 μg/ml.^29^ ATI levels in samples with different IFX/ATI levels status were then quantified by the four assays as described above.

### Statistical analyses

Categorical variables were described as absolute (*n*) and relative frequencies (%), and continuous variables were shown as the median and interquartile range (IQR). The quantitative agreement between assays could not be assessed because data was reported using different and arbitrary units (AU/ml). Therefore, the qualitative agreement of ATI levels or IFX/ATI levels status between pairs of assays was determined using Cohen’s kappa (*k*) coefficients and accuracy with 95% confidence intervals (CIs). The Cohen’s *k* coefficients were categorised according to the criteria of Landis and Koch: ⩽0.000 no agreement, 0.000–0.200 slight, 0.210–0.400 fair, 0.410–0.600 moderate, 0.610–0.800 substantial and 0.810–1.000 almost perfect agreement.^30^ Accuracy percentages of 0–4% were considered no accuracy, 4–15% minimal, 15–35% weak, 35–63% moderate, 64–81% strong and 82–100% almost perfect accuracy.^31^ Accuracy is the agreement between value found and an excepted reference value and the agreement refers to the closeness of two measured values, not to whether those values are correct or not (estimated by the kappa coefficient).

IFX/ATI levels status were stratified in four combinations of detectable (IFX+) or undetectable exogenous IFX and ATI-negative (ATI-) or ATI+ levels as follows: IFX+/ATI-, IFX+/ATI+, IFX-/ATI+, and IFX-/ATI-. To assess the impact of exogenous IFX concentrations on the quantification of ATI levels, graphical analyses plotted the mean of six measurements from six different samples (one measurement per sample), of ATI levels *versus* increasing exogenous IFX concentrations in spiked serum samples, by quantification assay, for each group of patients’ serum samples with low, intermediate, or high ATI+ levels. Statistical analysis was performed using SPSS version 24.0 (IBM Corp, Armonk, NY) and the graphical representation was performed using GraphPad Prism version 8.3.0 (GraphPad Software, Inc., San Diego, CA).

## Results

### Study population

This study analysed 200 serum samples collected from 57 IBD patients under IFX therapy. Table 1 shows the baseline demographic and clinical characteristics of the patients. Briefly, patients had a median age at diagnosis of 29 (19–36) years, 56.1% were female, 57.9% never smoked, 14.0% were current smokers, and 28.1% were former smokers. A total of 70.2% of patients had Crohn’s disease and 29.8% had ulcerative colitis; 22 patients (38.6%) were under concomitant immunosuppression (azathioprine or methotrexate).

**Table 1.** Characteristics of patients with IBD treated with infliximab.

	Patients (*n* = 57)
Age at diagnosis, median (IQR), years	29 (19–36)
Gender, *n* (%)	
Female	32 (56.1)
Male	25 (43.9)
Crohn’s disease, *n* (%)	40 (70.2)
Ulcerative colitis, *n* (%)	17 (29.8)
Smoking status, *n* (%)	
Never smoker	33 (57.9)
Former smoker	16 (28.1)
Current smoker	8 (14.0)
Concomitant IBD-related medication, *n* (%)	
None	21 (36.8)
Azathioprine	19 (33.3)
Steroids	9 (15.8)
Methotrexate	3 (5.3)
Oral 5-aminosalicylates	5 (8.8)
Time under biological therapy, median (min–max), months	6 (1–20)
IFX mg/kg, median (min–max)	6 (5–10)
Number of IFX received, median (min–max)	3 (0–12)
Dose intervals, median (min–max)	7 (5–8)
Dose optimization, *n* (%)	
No	47 (82.5)
Yes	10 (17.5)
Albumin g/l, median (min–max)	41.9 (29.3–66.4)

IBD, inflammatory bowel disease; IFX, infliximab, IQR, interquartile range; *n*, number of patients.

### Agreement for ATI+ levels

Qualitative agreement and accuracy of the QB rapid test and three established ELISAs was determined by quantifying the ATI levels in patients’ serum samples and stratifying the results into analytical and clinical cutoffs.

When stratified by analytical cutoffs for ATI+ levels (QB rapid test ⩾0.6 µg/ml, In-House 1.2 µg/ml, Theradiag 0.01 µg/ml, and Immundiagnostik 10 AU/ml), ATI+ levels were detected in 48 (24.0%) samples with the QB rapid test, 161 samples (80.5%) with In-House, 65 (32.5%) samples with Theradiag and 158 (79.0%) samples with Immundiagnostik. As shown in Table 2, a moderate agreement was found between the QB rapid test and Theradiag (*k* = 0.489), while a slight agreement was observed between the QB rapid test and In-House (*k* = 0.160) and QB rapid test and Immundiagnostik (*k* = 0.139). Comparisons between the remaining assay pairs revealed fair agreements (Table 2).

**Table 2.** Qualitative agreement between ATIs+ levels: comparison between assay pairs stratified by analytical and clinical cutoffs.

Assay comparison	Accuracy (95% CI)	Cohen’s kappa (95% CI)
**Analytical cutoffs** ^a^		
QB rapid test *versus* In-House	51 (44–57)	0.160 (0.102–0.217)
QB rapid test *versus* Theradiag	75 (69–81)	0.489 (0.384–0.595)
QB rapid test *versus* Immundiagnostik	43 (36–50)	0.139 (0.086–0.192)
In-House *versus* Theradiag	70 (63–75)	0.403 (0.301–0.505)
In-House *versus* Immundiagnostik	80 (73–85)	0.388 (0.235–0.541)
Theradiag *versus* Immundiagnostik	67 (60–73)	0.375 (0.276–0.474)
**Clinical cutoffs** ^b^		
QB rapid test *versus* In-House	49 (41–56)	0.163 (0.051–0.276)
QB rapid test *versus* Theradiag	85 (79–90)	–
QB rapid test *versus* Immundiagnostik	35 (29–42)	0.085 (0.000–0.177)
In-House *versus* Theradiag	34 (27–41)	–
In-House *versus* Immundiagnostik	72 (65–78)	0.289 (0.133–0.445)
Theradiag *versus* Immundiagnostik	20 (15–26)	–

a ATIs+ levels: ⩾0.6 μg/ml for QB rapid test, ⩾1.2 μg/ml for In-House, ⩾0.01 μg/ml for Theradiag, and ⩾10 AU/ml for Immundiagnostik.

b ATIs+ levels: ⩾1.7 µg/ml for QB rapid test, In-House and Theradiag, and ⩾10 AU/ml for Immundiagnostik.

ATIs+, anti-infliximab antibodies-positive; CI, confidence interval; QB, Quantum Blue.

Based on clinical cutoffs for ATI+ levels (QB rapid test, In-House, Theradiag ⩾1.7 μg/ml and Immundiagnostik > 10 AU/ml), QB rapid test detected 30 (15.0%) samples, Theradiag did not detect ATI+ samples, and In-House and Immundiagnostik detected the highest number of samples, 140 (70.0%) and 160 (80.0%), respectively. A total of 32% of the values negative with our threshold (<1.7 µg/ml) turn out positive with lower limit of quantification cutoff are under 0.010 µg/ml. In fact, these values might reflect only the intra-variability of the assay rather than represent the presence of antibodies. Although the In-House and Immundiagnostik assays detected an approximate number of ATI+ samples, not all samples matched. ATI+ levels were confirmed by both assays in 123 samples (61.5%). All 30 ATI+ samples identified by the QB rapid test were also positive in both the In-House and Immundiagnostik assays. As can be seen from Table 2, using the clinical cutoffs, a slight agreement was found between the QB rapid test and In-House (*k* = 0.163) or QB rapid test and Immundiagnostik (*k* = 0.085). The comparison of the In-House *versus* Immundiagnostik pair showed a fair agreement (*k* = 0.289). The *k* coefficient could not be calculated for the comparisons with Theradiag as this assay did not detect ATI+ samples.

### Agreement for trough IFX and ATI levels status

The accuracy and agreement of IFX/ATI levels status between pairs of assays were also evaluated. The patients’ serum samples were divided into four IFX/ATI levels status, using both analytical and clinical cutoffs, resulting in IFX+ concentrations in 90 (45.0%) or 80 (40.0%) of the 200 samples, respectively. The number of IFX/ATIs levels status for each assay in addition to the comparisons between tests can be assessed in Supplementary Table S1.

As shown in Table 3, considering the analytical cutoffs, the QB rapid test did not detect IFX+/ATI+ samples and Theradiag detected only one, corresponding to one of the IFX+/ATI+ samples detected by In-House. All assays were able to detect samples with the remaining IFX/ATI levels status. Overall, a strong accuracy was found between the In-House and the Immundiagnostik assays (80%) with a substantial agreement (*k* = 0.661). A strong accuracy was also found between the Theradiag and the QB rapid test (75%) or In-House assays (70%) with a substantial agreement (*k* = 0.625) or a moderate agreement (*k* = 0.531), respectively (Table 3).

**Table 3.** Qualitative agreement regarding the IFX/ATIs levels status: comparison between assays pairs stratified by analytical and clinical cutoffs.

Assay comparison	*n* (%)				Accuracy (95% CI)	Cohen’s kappa (95% CI)
	IFX+/ATIs+	IFX+/ATIs–	IFX–/ATIs+	IFX–/ATIs–		
**Analytical cutoffs** ^a^						
QB rapid test *versus* In-House	0 (0.0%)	42 (21.0%)	46 (23.0%)	3 (1.5%)	45 (39–53)	0.299 (0.211–0.388)
QB rapid test *versus* Theradiag	0 (0.0%)	89 (44.5%)	46 (23.0%)	15 (7.5%)	75 (68–81)	0.625 (0.535–0.715)
QB rapid test *versus* Immundiagnostik	0 (0.0%)	50 (25.0%)	46 (23.0%)	0 (0.0%)	43 (36–50)	0.275 (0.185–0.360)
In-House *versus* Theradiag	1 (0.5%)	42 (21.0%)	94 (47.0%)	2 (1.0%)	70 (63–76)	0.531 (0.433–0.629)
In-House *versus* Immundiagnostik	30 (15.0%)	22 (11.0%)	107 (53.5%)	0 (0.0%)	80 (73–85)	0.661 (0.568–0.753)
Theradiag *versus* Immundiagnostik	0 (0.0%)	49 (24.5%)	95 (47.5%)	0 (0.0%)	68 (61–74)	0.507 (0.407–0.606)
**Clinical cutoffs** ^b^						
QB rapid test *versus* In-House	0 (0.0%)	52 (26.0%)	30 (15.0%)	15 (7.5%)	49 (41–57)	0.343 (0.254–0.431)
QB rapid test *versus* Theradiag	0 (0.0%)	80 (40.0%)	0 (0.0%)	90 (45.5%)	89 (84–93)	0.808 (0.729–0.888)
QB rapid test *versus* Immundiagnostik	0 (0.0%)	41 (20.5%)	30 (15.0%)	1 (0.5%)	35 (29–42)	0.217 (0.138–0.297)
In-House *versus* Theradiag	0 (0.0%)	52 (26.0%)	0 (0.0%)	15 (7.5%)	34 (27–41)	0.219 (0.142–0.296)
In-House *versus* Immundiagnostik	13 (6.5%)	24 (12.0%)	105 (52.5%)	1 (0.5%)	72 (65–78)	0.531 (0.428–0.634)
Theradiag *versus* Immundiagnostik	0 (0.0%)	41 (20.5%)	0 (0.0%)	1 (0.5%)	20 (15–26)	0.129 (0.069–0.189)

a ATIs+ levels: ≥0.6 μg/ml for QB rapid test, ≥1.2 μg/ml for In-House, ≥0.01 μg/ml for Theradiag, and ≥10 AU/ml for Immundiagnostik. IFX+ levels: ≥0.4 µg/ml.

b ATIs+ levels: ≥1.7 µg/ml for QB rapid test, In-House and Theradiag, and ≥10 AU/ml for Immundiagnostik. IFX+ levels: ≥0.4 µg/ml.

ATIs, anti-infliximab antibodies; ATIs+, ATIs-positive levels; ATIs -, ATIs-negative levels; CI, confidence interval; IFX, infliximab; IFX+, IFX -positive levels; IFX -, undetectable IFX levels; QB, Quantum Blue; n, number of matching samples between assays for each IFX/ATIs status in a total of 200 samples.

Regarding the clinical cutoffs for the quantification of ATI levels, only the In-House and Immundiagnostik assays detected IFX+/ATI+ samples (Table 3). Comparing with the analytical cutoffs, the In-House and Immundiagnostik assays identified a similar number of samples in each IFX/ATI status. Conversely, the QB rapid test and Theradiag identified a higher number of IFX-/ATI- samples and a lower number of IFX +/ATI– samples. All assays were able to identify the remaining IFX/ATI status samples except for the Theradiag, which did not detect IFX–/ATI+ samples. An almost perfect accuracy was found between the pair QB rapid test and Theradiag (89%) with an almost perfect agreement (*k* = 0.808). The pair In-House and Immundiagnostik showed a strong accuracy (72%) and a moderate agreement (*k* = 0.531).

### Effect of exogenous IFX on ATI quantification

The impact of IFX on the quantification of ATI levels was evaluated by measuring spiked ATI+ serum samples (5, 10, 15, 30, 100 and 300 μg/ml IFX) with the four assays, based on the clinical cutoffs for ATI+ levels.

**Figure 1.**
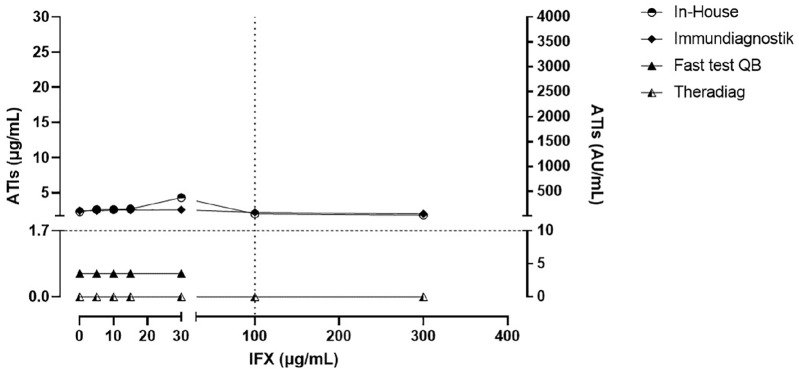
Low anti-infliximab antibodies levels (1.7–2.9 µg/ml) quantified by QB rapid test, In-House, Theradiag and Immundiagnostik assays in the presence of exogenous infliximab. The horizontal dotted line is the lower limit for positive levels of ATIs using the clinical cutoffs (1.7 μg/ml for QB rapid test, In-House and Theradiag, and 10 AU/ml for Immundiagnostik). The vertical dotted line is the upper limit of the therapeutic range of infliximab concentrations (0–100 μg/ml). ATIs, anti-infliximab antibodies; IFX, infliximab; QB, Quantum Blue.

Figure 1 displays the results in the samples with low ATI levels (1.7–2.9 µg/ml). No impact of exogenous IFX was evident in the Immundiagnostik assay. An IFX concentration of 30 µg/ml influenced the In-House assay by an additive concentration-effect; however, this influence was not evident at higher concentrations. In contrast, both the QB rapid test and Theradiag assays could not detect ATI+ levels (>1.7 µg/ml) in samples with all IFX concentrations. Moreover, the QB rapid test indicated invalid values in the samples containing exogenous IFX concentrations >30 μg/ml.

**Figure 2.**
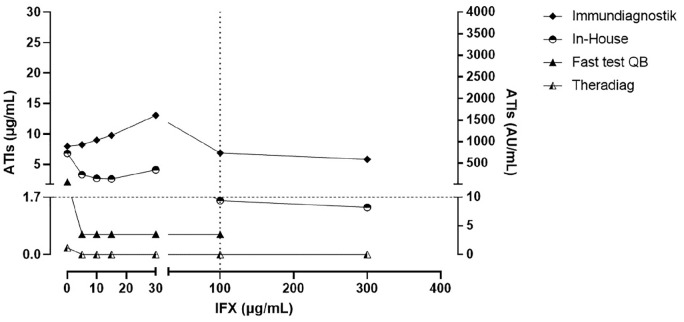
Intermediate anti-infliximab antibodies levels (3.0–9.9 µg/ml) quantified by QB rapid test, In-House, Theradiag, and Immundiagnostik assays in the presence of exogenous infliximab. The horizontal dotted line is the lower limit for positive levels of ATIs using the clinical cutoffs (1.7 μg/ml for QB rapid test, In-House and Theradiag, and 10 AU/ml for Immundiagnostik). The vertical dotted line is the upper limit of the therapeutic range of infliximab concentrations (0–100 μg/ml). QB rapid test indicated invalid values in some samples in the 100 µg/ml IFX concentrations. ATIs anti-infliximab antibodies; IFX, infliximab; QB, Quantum Blue.

Figure 2 presents the results in the samples with intermediate ATI levels (3.0–9.9 μg/ml). The impact of exogenous IFX was more evident in the Immundiagnostik and In-House assays with a decrease in ATI levels. In the presence of 30 μg/ml IFX, these assays were influenced by an additive concentration-effect, however, only the Immundiagnostik assay could detect ATI+ levels at the higher concentrations of exogenous IFX. The QB rapid test and Theradiag assays could not detect ATI+ levels with IFX concentrations from 5 to 30 µg/ml or with all concentrations, respectively. The QB rapid test indicated invalid values in samples containing IFX concentrations >100 µg/ml.

**Figure 3.**
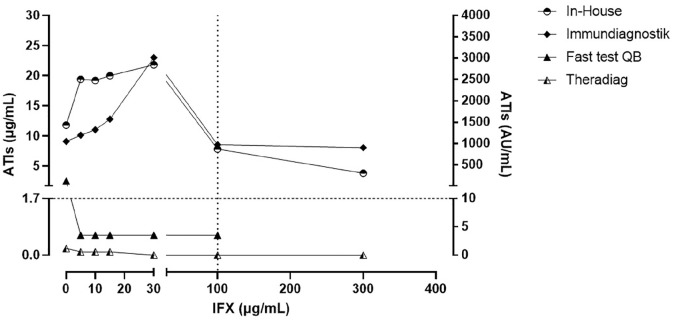
High anti-infliximab antibodies levels (⩾10 µg/ml) quantified by QB rapid test, In-House, Theradiag, and Immundiagnostik assays in the presence of exogenous infliximab. The horizontal dotted line is the lower limit for positive levels of ATIs using the clinical cutoffs (1.7 μg/ml for QB rapid test, In-House and Theradiag, and 10 AU/ml for Immundiagnostik). The vertical dotted line is the upper limit of the therapeutic range of infliximab concentrations (0–100 μg/ml). ATIs, anti-infliximab antibodies; IFX, infliximab; QB, Quantum Blue.

Figure 3 shows the results in the samples with high ATI levels (⩾10 μg/ml). The In-House and Immundiagnostik assays were able to detect ATI+ levels at all exogenous IFX concentrations. As described above, both assays showed an additive effect at 30 μg/ml IFX and ATI levels decreased at 100 μg/ml IFX. Similarly, the QB rapid test and Theradiag assays could not detect ATI+ levels in samples with IFX concentrations from 5 to 300 µg/ml or with all concentrations, respectively.

## Discussion

IFX is an effective therapy to the treatment of IBD.^32–34^ However many patients may lose response to treatment due to ATI.^35^ The ATIs measurement is crucial to adjust the therapy or switch to another drug. The most commonly used assays to evaluate ATI levels are ELISAs,^36^ which are very time-consuming. Therefore, the development of a rapid anti-IFX test allows a rapid quantification of ATIs, increasing the effectiveness of TDM and the immediate adjustment of the drug.^20,21^ In these present study, we evaluated and compared the qualitative agreement and accuracy of one rapid point-of-care test and three established ELISAs. Moreover, the impact of IFX on the quantification of ATI levels by the four assays was evaluated. IFX and ATI levels were measured in 200 serum samples from 57 IBD patients undergoing induction or maintenance therapy with IFX.

By using analytical and clinical-based cutoffs for defining ATI+ levels, we showed that the In-House and Immundiagnostik assays detected similar numbers of ATI+ samples with both cutoffs. On the other hand, the QB rapid test and Theradiag assays detected a higher number of ATI+ samples using the analytical cutoffs compared with the clinical ones. These results suggest a high prevalence of false negatives for the QB rapid test and Theradiag assays using clinical cutoffs. This finding is consistent with previous data obtained with the Theradiag assay.^17,27,37^ Several factors could explain these observations, such as drug interference underestimating ATI levels.^16,17,38^ The presence of IFX in the patient’s serum interferes with the binding of the marked IFX to the captured ATI, leading to false-negative results.^17,27,37^ The assays’ inability to detect ATI in the presence of IFX may render inconclusive test results.^17,27,37^

We next focused on the quantification of ATI levels in patients’ serum samples with different status for trough IFX and ATI levels (detectable or undetectable). We showed that the In-House and Immundiagnostik assays were more accurate and could detect ATI+ samples in the presence of IFX. However, in the presence of IFX, the QB rapid test did not accurately detect ATI+ levels using both analytical and clinical cutoffs. A kappa analysis to the IFX-/ATIs samples was also performed and the QB rapid test improves its capacity to detect ATIs in the absence of the drug (Supplementary Table S2). Furthermore, there was a disagreement between the QB rapid test and the In-House or Immundiagnostik assays in the quantification of ATI+ samples. These findings have clinical relevance and reinforces that the QB rapid test is affected by drug interference. Then, our results show that the QB rapid test and Theradiag measure only free ATIs s detecting a lower amount of ATI+ samples when compared with In-House Immundiagnostik assays. The ability to detect ATI in the presence of the IFX is important, as it was shown that IBD patients with both good IFX trough levels (⩾3 μg/ml) and ATI+ levels have significantly higher levels of C-reactive protein and less mucosal healing during treatment,^7,39^ which indicates a reduced control of inflammation mediated by these antibodies even when drug levels are adequate. Our results show that the disagreement increase when the samples had a double-positive or double-negative status, probably related to the specific limitations of each assay. This disagreement can also occur due to the cutoff point chosen to discriminate the ATIS positive from the ATIs negative. This led us to define two different approaches – clinical and analytical approach. The clinical approach seems to highlight the assays’ differences. Disagreement increased when samples had double-negative status, probably related to the fact that the QB rapid test detect a greater number of ATI- than the remaining assays. This disagreement could be explained due to the specific limitations and characteristics of each assay.

QB rapid test and Theradiag are drug-sensitive ATI assays, while In-House and Immundiagnostik are drug-tolerant ATI assays. Drug-sensitive ATI assays measures only free antibodies not bound to infliximab, detecting a lower amount of ATI+ samples when compared with drug-tolerant ATI assays. A recent study shows evidence that there is a different clinical interpretation of results when using drug-sensitive *versus* drug-tolerant assays.^40^ The choice of the cutoff to discriminate positive *versus* negative also enhances disagreement.

To better understand the impact of IFX on the quantification of ATI, we performed additional experiments using IFX- serum samples incubated with different concentrations of exogenous IFX. We were able to evaluate which IFX concentrations decreased each assays’ ability to quantify ATI+ levels. Notably, the addition of exogenous IFX concentrations corresponding to concentrations detected in clinical practice resulted in undetectable ATI levels by the QB rapid test. Using clinical cutoffs, this test could not detect ATIs in serum with intermediate (3.0–9.9 µg/ml) and high (⩾10 µg/ml) ATI+ levels in the presence of 5–300 µg/ml exogenous IFX concentrations. In contrast, the Immundiagnostik and In-house assays were slightly affected by the lowest concentrations of exogenous IFX. Also, these assays were able to detect ATI up to 300 μg/ml of IFX in serum with low, intermediate, and high ATI+ levels. We have previously described the same drug concentration dependency in these assays.^23^

These results show that the ATIs detected are affected by the drug. In this sense, our results show that the QB rapid test and Theradiag are drug-sensitive assays and the In-House and Immundiagnostik are drug-tolerant assays. Clinicians who use these data should have a general understanding of the assay methods to be able to interpret and implement the results. Therefore, these assays should not be interchangeably, and their results should not be directly compared.

The main limitation of this study was the measurement of ATIs levels performed on a single plate and only once, not allowing conclusions about inter and intra assays variability. Furthermore, all the ATI assays used in this study are non-functional assays (not detecting the neutralizing antibodies). In this study, patients were not followed up and it was not possible take conclusions about the relationship between the drug response and the ATI status. Moreover, with emerging reports on transient antibodies, it would be prudent to first ascertain the antibody persistence before making clinical decisions based on a single measurement of ATI levels.^41^ Further prospective studies with larger patient cohorts are needed to confirm and validate the findings of this study. Although the findings should be interpreted with caution, a key strength of this study is the large number of serum samples obtained from a multicentric and real-world heterogeneous cohort of IBD patients. Finally, the wide range of ATI+ levels allowed to evaluate the assays’ performance both at low and high levels. However, it is important to distinguish clinically between patients with ATIs < 3.7 µg/ml and >10 µg/ml, since patients with low ATIs levels are more susceptible to dose optimisation while patients with high ATIs levels usually require switch to another drug.

The findings of this study have several important implications for future practice. Clinicians should be aware that treatment optimisation may differ according to the assay used for TDM. The QB rapid test could not accurately detect ATI+ samples in the presence of IFX. In the optimization of the treatment, clinicians should be aware that the results of the different IFX/ATI status may differ according to the assays. The choice of the assay will probably have little influence on therapeutic decisions in the IFX+/ATIs– (change of drug class) and IFX–/ATIs+ status (change of anti-TNFα antibody drug), since agreement between assays is significantly higher in these circumstances. However, the agreement between assays was weaker when patients had double-negative (IFX–/ATI–) or double-positive (IFX+/ATI+) status. In these situations, erroneous therapeutic decisions may occur. Dose optimisation, shorter interval (IFX–/ATI–) and change of drug class or concomitant use of immunomodulators (IFX+/ATI+) should take into account the fact that the results are assay dependent. A reasonable approach to tackle this issue could be using the QB rapid test to quantify ATI levels only in IFX- samples, after performing another rapid point-of-care test to quantify the IFX levels in the patients’ serum samples.

In conclusion, we have shown that the QB rapid test can be used for the quantification of ATI levels in serum samples with undetectable IFX levels but should not be used in samples with IFX concentrations ⩾0.4 µg/ml. The comparison of qualitative agreements and accuracies between the QB rapid test and the In-House, Theradiag, and Immundiagnostik ELISAs suggest that these assays are not interchangeable for the quantification of ATI levels in IBD patients’ serum. These findings are particularly relevant for physicians when making clinical decisions about IBD treatment optimization based on ATIs quantification assays.

## Supplemental Material

sj-pdf-1-tag-10.1177_1756284820965790 – Supplemental material for Rapid test detection of anti-infliximab antibodies: performance comparison with three different immunoassays Supplemental material, sj-pdf-1-tag-10.1177_1756284820965790 for Rapid test detection of anti-infliximab antibodies: performance comparison with three different immunoassays by Cátia Rocha, Paula Lago, Samuel Fernandes, Luís Correia, Francisco Portela, Ana Isabel Vieira, Marta Patita, Bruno Arroja, Paula Ministro, Catarina Alves, Cláudia Camila Dias and Fernando Magro in Therapeutic Advances in Gastroenterology

sj-pdf-2-tag-10.1177_1756284820965790 – Supplemental material for Rapid test detection of anti-infliximab antibodies: performance comparison with three different immunoassays Supplemental material, sj-pdf-2-tag-10.1177_1756284820965790 for Rapid test detection of anti-infliximab antibodies: performance comparison with three different immunoassays by Cátia Rocha, Paula Lago, Samuel Fernandes, Luís Correia, Francisco Portela, Ana Isabel Vieira, Marta Patita, Bruno Arroja, Paula Ministro, Catarina Alves, Cláudia Camila Dias and Fernando Magro in Therapeutic Advances in Gastroenterology
