# Supplementary material for: Rapid test detection of anti-infliximab antibodies: performance comparison with three different immunoassays
Source: Ther Adv Gastroenterol. 2020 Nov 18;13:1756284820965790. doi: 10.1177/1756284820965790 (PMC7682213; doi:10.1177/1756284820965790)
Supplement: sj-pdf-1-tag-10.1177_1756284820965790 – Supplemental material for Rapid test detection of anti-infliximab antibodies: performance comparison with three different immunoassays [file sj-pdf-1-tag-10.1177_1756284820965790.pdf]

SUPLEMENTARY TABLES

Table 1 Number of IFX/ATIs levels for each test in addition to the comparisons between tests.

Assay	n (%)			
	IFX+/ATIs+	IFX+/ATIs-	IFX-/ATIs+	IFX-/ATIs-
**Analytical cutoffs[a]**				
QB rapid test	0 (0.0%)	90 (45.0%)	48 (24.0%)	62 (31.0%)
In-House	53 (26.5%)	37 (18.5%)	108 (54.0%)	2 (1.0%)
Theradiag	1 (0.5%)	89 (44.5%)	96 (48.0%)	14 (7.0%)
Immundiagnostik	50 (25.0%)	40 (20.0%)	110 (55.0%)	0 (0.0%)
**Clinical cutoffs[b]**				
QB rapid test	0 (0.0%)	80 (40.0%)	30 (15.0%)	90 (45.0%)
In-House	30 (15.0%)	50 (25.0%)	109 (54.5%)	11 (5.5%)
Theradiag	0 (0.0%)	80 (40.0%)	0 (0.0%)	120 (60.0%)
Immundiagnostik	41 (20.5%)	39 (19.5%)	119 (59.5%)	1 (0.5%)

ATIs, anti-infliximab antibodies; ATIs+, ATIs-positive levels; ATIs-, ATIs-negative levels; IFX, infliximab; IFX+, IFX-positive levels; IFX-, undetectable IFX levels; QB, Quantum Blue; n, number of samples for each IFX/ATIs status in a total of 200 samples.

[a]ATIs+ levels: ≥0.6 µg/mL for QB rapid test, ≥1.2 µg/mL for In-House, ≥0.01 µg/mL for Theradiag, and ≥10 AU/mL for Immundiagnostik. IFX+ levels: ≥0.4 µg/mL.

[b]ATIs+ levels: ≥1.7 µg/mL for QB rapid test, In-House and Theradiag, and ≥10 AU/mL for Immundiagnostik. IFX+ levels: ≥0.4 µg/mL

Table 2 Qualitative agreement between QB rapid test and the three established enzyme-linked immunosorbent assays (ELISAs) in the IFX negative samples/ATIs positive and negative samples

Assay comparison	n (%)		Cohen's kappa
	IFX-/ATIs+	IFX-/ATIs-	(95% CI)
**Analytical cutoffs[a]**			
QB rapid test versus In-House	46 (23.0%)	3 (1.5%)	0.040 (0.000-0.200)
QB rapid test versus Theradiag	46 (23.0%)	15 (7.5%)	0.204 (0.038-0.369)
QB rapid test versus Immundiagnostik	46 (23.0%)	0 (0.0%)	-
**Clinical cutoffs[b]**			
QB rapid test versus In-House	30 (15.0%)	15 (7.5%)	0.091 (0.000-0217)
QB rapid test versus Theradiag	0 (0.0%)	90 (45.5%)	-
QB rapid test versus Immundiagnostik	30 (15.0%)	1 (0.5%)	0.056 (0.000-0.017)

ATIs, anti-infliximab antibodies; ATIs+, ATIs-positive levels; ATIs-, ATIs-negative levels; CI, confidence interval; IFX, infliximab; IFX-, undetectable IFX levels; QB, Quantum Blue; n, number of matching samples between assays for each IFX/ATIs status in a total of 200 samples.

[a]ATIs+ levels: ≥0.6 µg/mL for QB rapid test, ≥1.2 µg/mL for In-House, ≥0.01 µg/mL for Theradiag, and ≥10 AU/mL for Immundiagnostik. IFX+ levels: ≥0.4 µg/mL.

[b]ATIs+ levels: ≥1.7 µg/mL for QB rapid test, In-House and Theradiag, and ≥10 AU/mL for Immundiagnostik. IFX+ levels: ≥0.4 µg/mL
