# Supplementary material for: Rapid test detection of anti-infliximab antibodies: performance comparison with three different immunoassays
Source: Ther Adv Gastroenterol. 2020 Nov 18;13:1756284820965790. doi: 10.1177/1756284820965790 (PMC7682213; doi:10.1177/1756284820965790)
Supplement: sj-pdf-2-tag-10.1177_1756284820965790 – Supplemental material for Rapid test detection of anti-infliximab antibodies: performance comparison with three different immunoassays [file sj-pdf-2-tag-10.1177_1756284820965790.pdf]

SUPPORTING INFORMATION

ANTI-IFX ANTIBODIES (ATIs) QUANTIFICATION ASSAYS

Quantum Blue®Anti-Infliximab quantification assay

This is a semi-quantitative sandwich immunoassay. Infliximab (IFX) fragments are conjugated to gold nanoparticles. On the test cassette the gold conjugate is released from a pad into the reaction system as the sample is applied. Anti-infliximab antibodies present in the sample will bind to the gold conjugate. Infliximab, specific for the polyclonal analyte, is immobilized on the test membrane and will capture the complex of gold conjugate and the ATIs, resulting in a colouring of the test line (T). This is described by the manufacturer's as a drug sensitive test (do not detect ATIs in the presence of drug and underestimate ATIs formation). Briefly, serum samples were diluted 1:10 and 80 µl of the diluted serum sample was loaded into the port of the test cassette. After 15 min of incubation, the cassette was read, and the results were shown on the Quantum Blue® Anti-Infliximab reader display. The reported concentrations are IgG equivalents (μg_{eq}/ml) to the monoclonal reference antibody used for standardisation. For the purpose of brevity, the results are thereafter expressed as µg/ml, rather than μg_{eq}/ml. The test information and calibration curve for each specific cassette lot was provided with a chip card to each test kit.

Immundiagnostik

This is a semifluid phase enzyme immunoassay (SFPE). The SFPE uses an initial acid buffer treatment (peroxidase labelled therapeutic antibody) and the biotinylated therapeutic antibody to dissociate the ATIs from the therapeutic

antibody in order to acquire free ATIs. Acidified samples bind via biotin to the streptavidin coated microtiter plate. It is detected via the peroxidase conjugate with the peroxidase converting the substrate tetramethylbenzidine (TMB) to a blue product. This is described by the manufacturer's and in the literature as a drug tolerant test (assay sensitivity in the presence of drug). The enzymatic reaction is stopped by adding an acidic solution. Samples' absorbance was read at 450/620 nm and the results were expressed as AU/ml. The results are interpreted using the cut-off control (10 AU/ml) and the samples which have a higher average optical density (OD) than the cut-off control are positives. According to the manufacturer, the lower limit of measurement range is the Limit of blank (LoB).

Theradiag

A classical bridging enzyme-linked immunosorbent assay (BE) unable to measure ATIs in the presence of drug[21,22]. The BE uses a double-antigen bridge: ATIs create a bridge between IFX immobilized on the plate and IFX enzyme-linked conjugate. This is described by the manufacturer's as a drug sensitive test. Samples' absorbance was read at 450 nm and the results were expressed as ng/ml. If the samples presented higher values than the upper limit (200 ng/ml) of the kit, this was considered as the result. The lower limit of quantification was 10 ng/ml. For the purpose of comparisons, the results are thereafter converted and expressed as μg/ml.

In-house

The In-house is a sandwich enzyme-linked immunosorbent assay (ELISA) that uses antihuman lambda chain (AHLC) conjugated antibody in the detection phase, taking advantage of the fact that IFX is composed of kappa chains[17]. Briefly, IFX was added to a plate precoated with tumour necrosis factor alpha (TNFα) (Peprotech, Rocky Hill, NJ, USA). This is described in the literature as a drug tolerant test. The serum samples were then diluted (1:50) and incubated for 60 min, at room temperature. After four washes, a Goat anti-human lambda chain HRP-labelled antibody (Serotec, Oxford, UK) was added and incubated for 60 min, at room temperature. Afterwards, TMB (Millipore, MA, USA) substrate was added, and the reaction was stopped 6 min later with 2 mol/l H_2SO_4. Lastly, samples' absorbance was read at 450/540 nm and the results were expressed as µg/mL (µg/mL) after normalisation against results obtained using a standard curve of goat anti-human F(ab')2 fragment antibody (MP Biomedicals). For the purpose of brevity, the results are thereafter expressed as µg/mL, rather than µg/ml-e. The lower limit of quantification was 1.2 µg/mL.
